# Integrating competency analysis into national rehabilitation workforce evaluation: a case study

**DOI:** 10.1186/s12960-023-00843-3

**Published:** 2023-08-23

**Authors:** Jody-Anne Mills, Weronika Krzepkowska, Alarcos Cieza, Paloma Cuchi, Pascal Zurn, Stephanie Doris Short, James W. Middleton

**Affiliations:** 1 Department of Noncommunicable Diseases, World Health Organization, Avenue Appia 20, 1211 Geneva 27, Switzerland; 2 World Health Organization Country Office, Poland, Al. Jerozolimskie 155, 02-326 Warsaw, Poland; 3 Health Workforce Department, World Health Organization, Avenue Appia 20, 1211 Geneva 27, Switzerland; 4 Faculty of Medicine and Health, WHO Collaborating Centre for Strengthening Rehabilitation Capacity in Health Systems, The University of Sydney, Sydney, Australia; 5 Faculty of Medicine and Health, John Walsh Centre for Rehabilitation Research, The University of Sydney, Sydney, Australia

**Keywords:** Health workforce evaluation, Rehabilitation workforce, Competency analysis

## Abstract

**Background:**

Establishing a workforce capable of meeting population needs is contingent on evaluation that can inform sound policy and planning. Health workforce evaluation has traditionally relied on health labour market analysis and workload estimations. To date, competency analysis has not been included in national health workforce evaluation, despite that fact that the findings may go far in guiding decisions around workforce composition, optimisation and education and training. This case study sought to assess the feasibility and perceived added value of integrating competency analysis into national rehabilitation workforce evaluation, and to determine how competency analysis can shape rehabilitation workforce planning. The findings of the case study can be used to explore the integration of competency analysis in the evaluation of other health-related occupational groups.

**Methods:**

Participant observation was complemented by key informant interviews with experts engaged in the national rehabilitation workforce evaluation in Poland. These experts represented stakeholders in policy, education, research, clinical practice and professional associations.

**Results:**

The results indicated that competency analysis can be feasibly integrated into national rehabilitation workforce evaluation, particularly when implementation is supported through the use of online platforms. However, the collection of additional data using other tools, such as a survey of the behaviours and tasks of a wider sample of rehabilitation workers, could strengthen data reliability. Experts perceived findings of the competency analysis to be valuable for expanding the understanding of rehabilitation, shedding light on task allocation and deployment of the existing rehabilitation workforce, and advocating for the rehabilitation workforce to be strengthened, especially in relation to those occupations which may not be recognised or valued as rehabilitation workers. Although it was not possible to fully explore the impact of competency analysis data on rehabilitation workforce planning and development in this study, experts suggested that its availability would likely foster greater cooperation among occupations, which has been missing at the policy and planning level to date. It further demonstrates what competency data should be collected and reported, and provides richer information to guide decisions.

**Conclusions:**

Competency analysis complements traditional labour market analysis and workload estimates, adding depth to the understanding of how members of the workforce perform and perceive themselves, and how deficiencies in the workforce impact on the provision of care to specific population groups.

## Introduction

In 2021, the World Health Organization launched the *Rehabilitation Competency Framework* (RCF), which describes the competencies and activities of the rehabilitation workforce [1]. The framework is relevant to all occupations that deliver care that aims to optimise functioning and reduce disability. It crosscuts specialisations and settings, and is, therefore, capable of being adapted and adopted for application in any specific context [2]. In its original form, the RCF provides a unique opportunity to conduct a competency analysis of the rehabilitation workforce. This has not previously been possible due to the absence of: (1) a shared description of proficiency levels that spans the spectrum of qualifications and expertise encompassed within the rehabilitation workforce; and (2) a comprehensive list of tasks that indicate the scope of rehabilitation care delivered by the rehabilitation workforce collectively. The RCF addresses both these barriers, opening the door to a new level of workforce evaluation extending beyond that of traditional health labour market analysis (HLMA).

Box 1. The rehabilitation workforce The rehabilitation workforce comprises a diverse group of occupations which provide interventions that aim to maximise functioning. They address needs associated with impairments in communication, mobility, cognition and mental health to enable people to be as independent as possible in daily activities and maximise their participation in education, work and meaningful life roles [3]. The rehabilitation workforce includes audiologists, occupational therapists, physiotherapists, physical and rehabilitation medicine doctors, prosthetists and orthotists, and speech and language therapists, as well as others whose scope of practice is either solely or partially dedicated to delivering rehabilitation.

Despite their unique and important contribution to health, the rehabilitation workforce lags behind other health occupations in its development and is grossly underfunded and under-prioritised in many health systems [4–7].

While HLMA is well-recognised as integral to sound policy and planning, it does not generate information about the proficiency of the workforce across key areas of performance, such as clinical practice, research and leadership, the tasks that workers perform or the  allocation of tasks across occupations. These kinds of data are valuable for identifying opportunities for performance improvement and workforce optimisation among educators, regulators and professional associations, amongst others. The data from competency analysis are also highly relevant in the context of a developing workforce, where educational capacity is emerging and curricula are being refined, and where rational task allocation is critical to ensuring that essential care is available to as many people as possible.

The rehabilitation workforce, and the health workforce more broadly, face numerous barriers, such as critical shortages, high rates of unemployment in some occupations, and issues of quality and access. These challenges pose a major barrier to the achievement of Sustainable Development Goal 3, particularly the provision of universal health coverage, as services cannot be delivered without a skilled workforce [7–9]. These challenges have been amplified globally by the impact of the COVID-19 pandemic [10]. The prevalence of health (and rehabilitation) workforce challenges reflects the complexity of their underlying causes, and the financial investment needed to address them. For this reason, policy makers, educators, regulators and professional associations need to be equipped with rich and meaningful data to guide policy development, strategic investment and effective action. As shown in Table 1, the information available through competency analysis could complement that generated in HLMA to better guide policy, planning and development. Furthermore, the combination of HLMA and competency analysis data enables more explicit links to be drawn between the state of the workforce and the delivery of health care, making a more compelling case for investment.

**Table 1:** Data generated through health labour market analysis and competency analysis

Health labour market analysis	Competency analysis
• Population health needs and subsequent workforce requirements • The supply of workers • The demand for workers, i.e., the number of paid job posts • The absorption of workers into health services • Labour market failures, such as a mismatch between supply and demand • The feasibility and impact of potential actions	• Proficiency profile, or level of performance of each occupational group in key areas of performance • Task allocation across occupational groups, i.e., which occupations perform which assessments and interventions, and the proportion of the workforce who are confident in performing the assessment or intervention
Combined data	
• The implications of the workforce situation for patient care, i.e., what a deficiency in the availability of an occupation means for the care of people with a specific health condition

Recognising that the rehabilitation workforce lags behind other health-related occupational groups in its development and remains under-funded and poorly prioritised, the World Health Organization developed guidance, tools and a methodology for conducting a national rehabilitation workforce evaluation [11, 12]. This suite of resources, collectively termed the Guide for Rehabilitation Workforce Evaluation (GRoWE), combines a HLMA approach with competency analysis grounded in the RCF with the aim of developing a national rehabilitation workforce report and action plan. GRoWE was piloted to assess the practicality of the approach, identify any technical issues with the tools, and determine the benefit of integrating competency analysis in national rehabilitation workforce evaluation in light of the additional time and data analysis required. This study presents the findings of the GRoWE pilot in Poland, using observation and key informant interviews to address three questions:

1. Is it feasible to integrate competency analysis into national rehabilitation workforce evaluation?
2. What is the perceived added value of integrating competency analysis into national rehabilitation workforce evaluation?
3. How can the findings of competency analysis shape rehabilitation workforce planning?

The findings of the study will be used to inform the final form of GRoWE and guide its implementation in other countries.

Box 2. Competency analysis within GRoWE The competency analysis within GRoWE has two components: a proficiency profile and a task mapping exercise. Both are conducted by samples (typically 3–5 workers) from each occupation, who are identified by the project officer with assistance from national professional associations when these exist. The proficiency profile and task mapping are performed either in-person or virtually, or using combination of these, and are conducted in the native language of the workers. The GRoWE workbook is translated when needed. Guidance is provided by the project officer to the degree requested by the workers.

**Proficiency profiling**

**Fig. 1:**
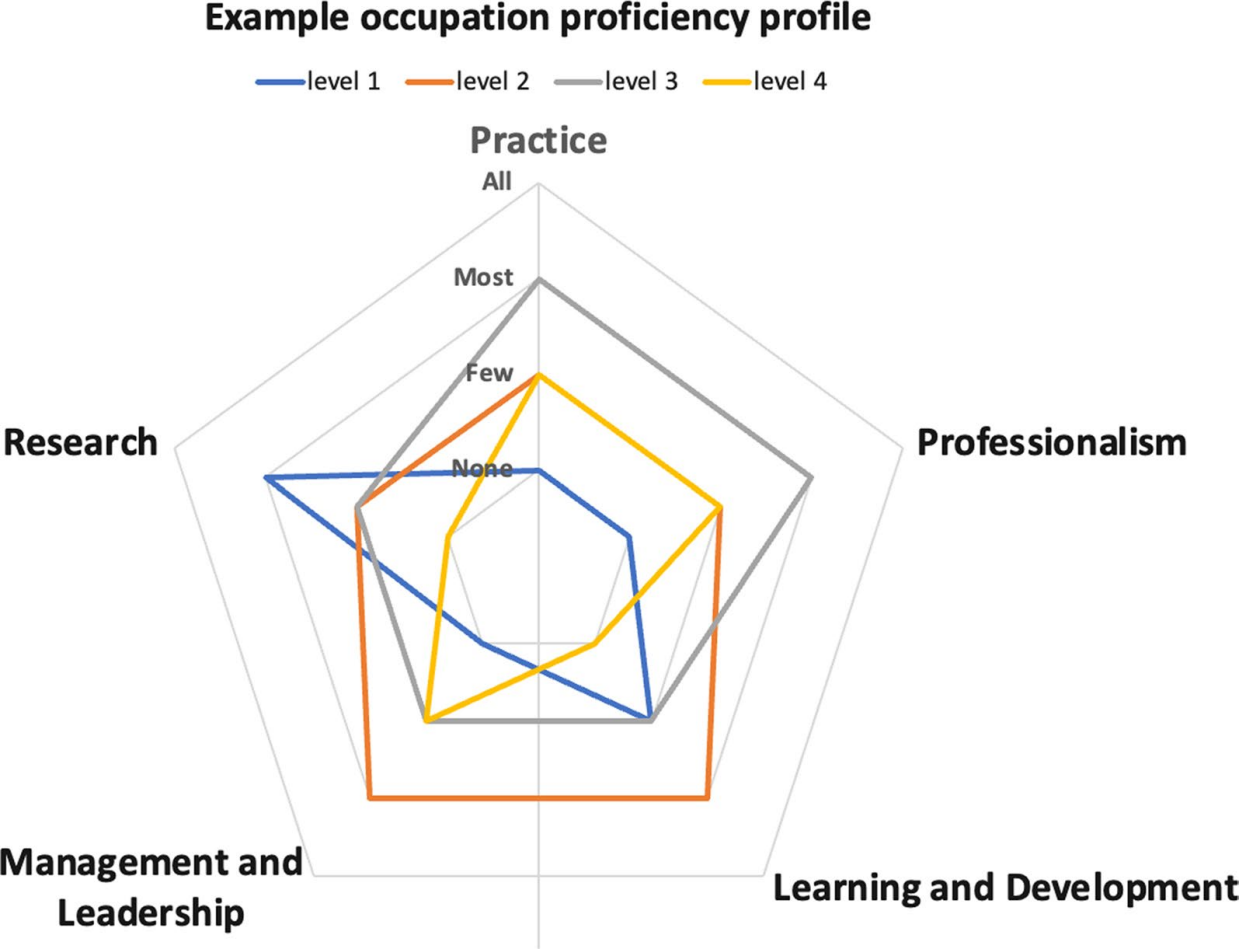
Example of proficiency profile for a rehabilitation occupational group

Proficiency profiling aims to provide an overview of the proficiency of an occupation across the five domains of the RCF: Practice, Professionalism, Learning and Development, Management and Leadership, and Research. It does so by requesting a sample of workers from the occupation to use an Excel-based tool to indicate the proportion of their occupation (none, few, most or all) that aligns with different levels of proficiency. These levels reflect a progression in autonomy, decision-making responsibility, and specialisation from level 1 to level 4. A description of a worker at each level is provided for each of the RCF domains. For example, the physiotherapists completing the exercise may report that, for the Practice domain, none of their occupation is at level 1, a few are at levels 2 and 4, and most are at level 3. Importantly, the assignment of proportions to the four levels can be different for each domain, thereby identifying areas needing to be strengthened in pre- or post-service education and training. The proficiency profiling exercise generates a spider-graph for each occupation, as shown in Fig. 1.

**Task mapping**

Task mapping aims to describe how essential rehabilitation tasks (assessments and interventions) are allocated across the occupations. The task mapping exercise identifies which tasks are never or rarely delivered based on the availability of the occupation to whom they are allocated. It can also show which tasks are being delivered by multiple occupations, exposing potential areas for further optimising the workforce and improving efficiency. Like the proficiency profiling exercise, the task mapping exercise is performed by a sample of workers from each occupation included in the evaluation. The exercise is conducted using an Excel-based tool in which participants indicate the proportion of their occupation that would be confident to deliver each assessment and intervention. The assessments and interventions included in the exercise are those relevant to a number of health conditions, including stroke, spinal cord injury, cerebral palsy, amputation, musculoskeletal conditions (lower back pain, neck pain, arthritis, and fracture), chronic obstructive pulmonary disease, acute myocardial infarction, hearing impairment, and schizophrenia. The conditions are drawn from the WHO Package of Interventions for Rehabilitation, and were selected based on their prevalence or incidence, their impact on functioning, and their amenability to rehabilitation.

The task mapping data from each occupation is compiled in one large table for ease of review, while a separate table shows how the task allocation, combined with the availability of each occupation, impacts the delivery of rehabilitation care for people with each health condition. An overview of the findings of the task mapping and key conclusions of the competency analysis of the rehabilitation workforce in Poland are presented in the Annex (note that this does not include the raw data from either the proficiency profiling or task mapping).

## Methods

### Study design

This study employed participant observation methodology, whereby two researchers (authors JM and WK) were immersed in the pilot, enabling them to interact with the GRoWE resources and experience each step of implementation. The authors further observed the time taken to complete the different competency analysis exercises, the challenges experienced by the stakeholders completing the exercises, how the findings were reported and how they were interpreted and applied in rehabilitation workforce planning.

Key informant interviews were also conducted with experts engaged in the evaluation, including those working in policy, education, professional associations and clinical practice. Lines of enquiry explored stakeholders’ experiences in identifying and reporting data, their perception of the findings and their added value, as well as their recommendations for future evaluations.

### Study population

This study draws on the insights of the following stakeholders:

- The WHO personnel overseeing the development and piloting of GRoWE (JM)
- The project officer responsible for implementing GRoWE in Poland (WK)
- Experts engaged in the evaluation (n = 5). The demographic profile of the experts is presented in Table 2.

**Table 2:** Demographics of key informants (experts) and their contribution

Expert code	Gender	Professional Role	Contribution to the evaluation
KI1	F	Professor, Faculty dean	Provided data, participated in workshops, reviewed draft report
KI2	M	Professor, hospital management (and parent of a child with a disability)	Participated in workshops, reviewed draft report
KI3	M	Past president of a professional association, practitioner, researcher	Completed analysis exercises, reviewed report, participated in workshops
KI4	M	Leader in a professional association, practitioner	Provided data, reviewed report, participated in workshops
KI5	F	National Institute of Public Health (policy), practitioner, researcher	Provided data, reviewed report, participated in workshops

### Recruitment

The GRoWE project officer (WK) disseminated an invitation to participate in feedback interviews via email to the experts who had engaged in the evaluation. The email included a participant information sheet (translated into Polish) and a link to a survey which showed the times available for interviews, and asked if interpretation would be needed. Participants interested in being interviewed were directed to complete the form and contact the WHO focal point (JM), who provided a consent form (also translated into Polish) and confirmed the time of the interview.

The relatively small sample of key informants partly reflects the challenges associated with recruiting individuals who are typically time-poor as well as the size (n = 42) of the pool of potential interviewees (i.e., experts who had participated in the evaluation). Nonetheless, the sample represented a wide range of perspectives, including policy makers, researchers, educators, practitioners, and leaders of professional associations. Finally, the themes emerging from analysis of the data achieved saturation, suggesting that additional interviews would not have generated new insights.

### Data collection

Key informant interviews were conducted by the WHO focal point and were recorded and transcribed using Microsoft Teams. Interpretation was offered to all key informants, but was only partially used by two, predominantly when technical language was being used. Transcripts were manually cleaned for analysis. Data from the participant observation were gathered through a systematic debriefing between the WHO focal point (JM) and GRoWE project officer (WK). The debrief involved detailed, goal-focused discussion, framed around the topics of engagement, feasibility, perception, added value and additional lessons learned. The data from the debrief were compared with the findings from the key informant interviews and the results of the competency analysis. These triangulated data were discussed by all authors of this paper.

### Data analysis

Thematic analysis of the interview transcripts was performed using NVIVO software. Results of the thematic analysis were reviewed and agreed by all authors and compared with observational findings.

### Ethical approval

Ethical approval for this study was obtained from the Human Research Ethics Committee of The University of Sydney, Australia (project number 2021/824).

## Results

Analysis of the data from the key informant interviews and project officer debrief revealed several themes related to the perceived value of the competency analysis, as well as of the evaluation more broadly. Themes also emerged around challenges experienced with data collection and quality, and several experts offered suggestions for strengthening future implementation of the evaluation. The main themes are elaborated below. Note that key informants and the project officer are referred to as “experts”.

### Working with the concept of a rehabilitation workforce

GRoWE was designed as a resource for the rehabilitation workforce collectively, rather than for any specific occupation. This was reflected in the competency analysis in particular, which presented rehabilitation assessments and interventions targeting a range of areas of functioning (e.g., mobility, communication, respiration, mental health.). Experts reported that participating in an evaluation that adopted the concept of a “rehabilitation workforce” challenged the identity of some occupations and expanded stakeholders’ understanding of what rehabilitation is and what its workforce comprises. For example, many speech and language therapists, who in Poland work primarily in the education sector, had not considered themselves as health workers or as rehabilitation workers. Conversely, psychologists in Poland had been working hard to promote themselves as rehabilitation professionals, but had encountered challenges due to the narrow perception of rehabilitation in the country. Experts noted that rehabilitation is largely synonymous with physiotherapy and rehabilitation medicine in Poland, and that other occupations, especially those which do not primarily address ‘physical’ impairment, are overlooked. The GRoWE project officer noted, “There are two queens or kings—doctors and physiotherapists—and the rest are underestimated”. GRoWE challenged this perception, placing equal importance on the competencies and tasks of each occupation included in the evaluation.

### Identifying and managing scopes of practice

The competency analysis was reported by experts to be valuable in addressing the issue of informal task sharing. Task sharing was noted to be widespread among the rehabilitation workforce in Poland, especially in relation to physiotherapists absorbing tasks from the scopes of practice of occupational therapy and speech and language therapy. Task sharing was considered to be necessary in Poland given the underdevelopment of many of the rehabilitation occupations but was not always well-supported with education and training. Furthermore, it was suggested, physiotherapists tended to assume that they were responsible for all or most rehabilitation tasks—a belief that likely stems from the absence of other occupations in many settings. As one expert reported, “we need to make the space for [other occupations], because sometimes physiotherapists here in Poland are thinking that they are all in one, but we are not” (KI5).

The competency analysis was considered by experts to be useful in showing which occupations were working at what level and what tasks were being performed by whom. This was seen as central to ensuring “proper use of available workforce” (KI2). Despite some limitations in the competency analysis (see data collection and quality below), the results were seen by experts as an important starting point for discussion and as the basis for the development of more comprehensive competency analysis tools.

### Multidisciplinary collaboration

A perceived benefit of GRoWE in general and the competency analysis exercises in particular was its ability to foster collaboration between the different rehabilitation occupations. Experts noted that, although some occupations were accustomed to working together in the clinical setting, this rarely translated into cooperation in workforce evaluation and planning. Completion of the exercises necessitated teamwork, while the workshops brought all occupations together around a virtual table, with a common aim. Experts noted that this had previously been lacking. One expert stated, “I was missing this openness and the willingness to cooperate between the different sections of the rehabilitation system. Therefore, it was perfect for me” (KI5).

### Data collection and quality

All experts noted challenges with data collection and quality. These related to all aspects of the evaluation, but reliability was specifically flagged as a concern for the competency analysis. This concern was linked to the subjective nature of the competency analysis exercises, which relied on a small sample of workers reporting on behalf of their wider occupation. The project officer reported that some stakeholders felt uncomfortable with the responsibility of representing their workforce. For example, when completing the competency analysis exercises, participants reported tension between responding based on what is expected of the occupation and the actual performance of workers. While the instruction for the exercises was to report actual performance, it was not always possible for the sample of workers completing the exercise to gauge this. Concerns about reliability were greater for unregulated occupations, such as occupational therapy, for which there is considerable variability in levels of proficiency and scopes of practice. Given these concerns, experts felt that the results of the competency analysis should be interpreted as preliminary and used to facilitate further investigation. It was also suggested that a survey instrument and/or focus groups could be used in addition to the exercises to obtain a wider range of perspectives and opinions, thereby strengthening the reliability of the data.

As the GRoWE pilot in Poland took place in the context of the COVID-19 pandemic, stakeholders had to meet virtually, and data collection occurred via emails, phone calls and online meetings and workshops. This format was considered by the experts to have several benefits, including greater flexibility and the ability to engage stakeholders from different geographic regions of the country. The GRoWE project officer noted however, that face-to-face discussion among stakeholders, even in a single workshop, had the potential to strengthen stakeholder engagement with the evaluation. Meeting in person was thought to enable better communication and connection among stakeholders (especially those who had not known each other prior to the evaluation) and between stakeholders and the project officer, to whom data were reported. Experts tended to agree that a hybrid approach would be optimal, with online communication complemented by at least one in-person workshop.

## Discussion

Traditionally, health workforce evaluation has focused on labour market analysis. The present study is the first to examine how competency analysis can complement labour market analysis in the context of national workforce planning for rehabilitation. To date, the application of competency analysis has been limited to the evaluation of specific workforce contexts, such as emergency preparedness or optimising the nursing and midwifery workforce in low-resource countries. Specifically, this study aimed to assess the feasibility, perceived added value of integrating competency analysis into national rehabilitation workforce evaluation, and its ability to shape rehabilitation workforce planning. These questions were examined in the context of the pilot of GRoWE in Poland, during which participant observation was conducted by the GRoWE project officer and the WHO focal point, and key informant interviews were conducted with experts engaged in the evaluation. Analysis of the triangulated data indicated that such integration was feasible and that it added considerable value to the rehabilitation workforce evaluation.

### Feasibility

The expansion of platforms for online working and the increased familiarity with remote working associated with the COVID-19 pandemic enabled GRoWE to be implemented virtually. This greatly enhanced its feasibility, allowing stakeholders from different parts of the country to engage and minimising the disruption to their regular work. However, a hybrid approach, whereby at least one workshop is held in person, is likely to be optimal as it will provide an opportunity to complete more challenging analysis exercises in the presence of the project officer. In-person workshops will further strengthen GRoWE’s role in fostering relationships among stakeholders, especially those from different occupations, and build greater rapport between the stakeholders and project officer, which may help to sustain their participation throughout the evaluation. Such a hybrid approach was adopted with great success in other pilots of GRoWE that were conducted in Rwanda and Nepal after the pilot in Poland had commenced. These additional pilots also demonstrated that the ideal balance between in-person and virtual working is context-dependent and is influenced by what is considered culturally appropriate and the availability and uptake of the required technology.

The method of using a small representative sample of workers from each occupation was initially adopted by GRoWE to address the challenges associated with coordinating large groups of people to complete shared exercises and achieve consensus. Although this approach enhanced the feasibility of the competency analysis, it reduced the reliability of the data for occupations with a large workforce. This may not be of concern when GRoWE is implemented in countries with a much smaller supply of rehabilitation workers, where a sample of 5 workers may represent 25% or more of the occupation. When this is not the case, the use of additional data collection instruments to obtain input from a wider range of perspectives would be valuable. A survey instrument could be developed to complement the existing analysis exercises and serve to verify their findings.

In summary, the use of virtual meeting platforms and the ability to use samples of workers to represent each occupation made the integration of competency analysis in Poland’s evaluation of the rehabilitation workforce feasible and suggests that it could be used in other countries and possibly with other occupational groups. However, the resource requirements and logistical burden of conducting the competency analysis exercises need to be acknowledged, and necessitate a commitment to using the findings to further develop the workforce.

### Perceived added value

In this case study, the competency analysis complemented traditional labour market data in several ways. First, from a practical perspective, the competency analysis data provided information on task allocation and worker proficiency that can guide action in education and inform decisions and practices around task sharing, which are critical in a country without an adequate supply of workers. Second, through participating in the competency analysis exercises, stakeholders became aware of the concept of a rehabilitation workforce and the broader scope of rehabilitation interventions. As such, the competency analysis exercises served as mechanisms for education and advocacy. This was particularly significant for occupations, such as occupational therapy, speech and language therapy and clinical psychology, who are less likely than physiotherapists or physical and rehabilitation medicine doctors to be seen or valued as rehabilitation workers, despite their unique and critical contributions. Third, the competency analysis identified the kinds of data that are important to collect and report, or routinely explore. This may help to improve future data collection practices beyond the evaluation itself.

### Impact on rehabilitation workforce planning

It remains to be seen what impact the competency analysis data will have on rehabilitation workforce planning in the months and years ahead. However, experts believed that both the process and results of the analysis will strengthen planning and development by: fostering cooperation between different occupations, which has heretofore rarely occurred at a policy and planning level; identifying the types of data that are important to collect and report; and providing information about the allocation of tasks and proficiency of workers. The latter may also help guide the design and delivery of rehabilitation workforce education in the country by highlighting possible areas of underperformance and areas for interdisciplinary training to facilitate task sharing.

## Limitations

This study captured the findings from only one pilot of GRoWE, which was conducted in a high-income country. However, the feasibility of the competency analysis, the extent to which it is valued and its impact on planning may differ from country to country. The results of this pilot should, therefore, be interpreted with caution, and lessons should continue to be drawn from the further implementation of GRoWE in future.

## Conclusion

Competency analysis appears to be a valuable addition to labour market analysis for evaluating the rehabilitation workforce. The approach is of potential interest to other health-related occupational groups, such as the vision or hearing workforce, which comprise a number of occupations that make unique but interrelated contributions to patient care. Competency analysis in the context of national workforce evaluation is a novel practice and, while it is an exciting innovation, its application should be approached with an openness to learning and adaptation.

## Data Availability

Raw data from the key informant interviews are confidential and will not be disclosed. Raw competency data from the case study may be released at the Polish Ministry of Health’s discretion. The Guide for Rehabilitation Competency Framework, is available via https://www.who.int/teams/noncommunicable-diseases/sensory-functions-disability-and-rehabilitation/guide-for-rehabilitation-workforce-evaluation. Contact millsj@who.int for enquiries regarding data and material access.

## Annex

### Overview of task mapping findings and key conclusions of the competency analysis

#### Overview of findings from the task mapping

##### Gaps in the delivery of rehabilitation assessments and interventions

The task mapping identified potential gaps of concern in the assessment of:

- cognitive impairment
- swallowing and salivation impairment
- hearing impairment
- fatigue
- performance of activities and participation in the home, education and work environment.

Gaps were also identified in the ability to deliver the following rehabilitation interventions:

- environmental modifications to home, school and workplace
- educating and supporting teachers, classmates and employers
- airway clearance techniques
- community mobility interventions (e.g., navigating transportation)
- speech and language interventions
- interventions for pain
- interventions for sexual functions and intimate relationships
- interventions for addressing activities and participation, such as self-care interventions and vocational training.

Gaps were further identified in provision and training in the use of assistive products for sensory impairments, such as:

- hearing aids
- Braille displays and writing equipment
- screen readers
- gesture to voice technology
- deaf-blind communicators and communication software.

These assessments and interventions are considered essential in the rehabilitation care of persons with health conditions that are widely prevalent and/or have a profound impact on functioning. As such, their absence is associated with significant unmet needs, resulting in reduced functioning and participation in education, work and community life.

##### Overlaps in the delivery of rehabilitation assessments and interventions

It is not uncommon, nor necessarily problematic, for there to be a degree of overlap in the provision of certain rehabilitation assessments and interventions. However, overlaps in scopes of practice that develop unintentionally over time can, if not systematically considered, result in inefficiencies. In other instances, it may be beneficial to introduce further overlap in scopes of practice to compensate for a particularly severe shortage of an occupational group.

In this study, the identification of overlaps was complicated for two main reasons. First, prosthetists and orthotists in Poland come from a range of backgrounds, and thus do not have a uniform scope of practice. Second, the profession of occupational therapy is still unregulated, again resulting in variability in scopes of practice. Nevertheless, the task mapping identified several areas of overlap that warrant exploration, as well as interventions that could potentially be expanded into an alternative occupation’s scope of practice to ensure they are available to those who need them.

Existing overlaps warranting exploration include, but are not limited to:

- mobility assessments
- assessment of the environment (home, school and workplace)
- the provision of mobility aids (such as wheelchairs, crutches, walking canes and walking frames), grab bars and compression garments.

#### Key conclusions drawn from the findings of the competency analysis

1. There are competency gaps in the rehabilitation workforce, particularly in the domains of Learning and Development, Management and Leadership, and Research. This indicates scope for strengthening in education and training.
2. There is inconsistency in competency levels both within and between rehabilitation occupational groups, reflecting differences in education and training and inadequate development and verification of competency standards.
3. There is a lack of evidence-based practice in the rehabilitation workforce, driven by a lack of investment in research skills, lack of access to and poor use of clinical guidelines and protocols, and limited mechanisms for knowledge sharing and promotion.
4. Rehabilitation workers lack adequate access to supervision, support and mentorship, including from peers, particularly for those working in more remote contexts. This hinders the development of the emerging workforce and potentially compromises patient safety.
5. There are inadequate opportunities for sub-specialisation, particularly for occupational therapists, which limits the ability of the rehabilitation workforce to meet more complex patient needs.

## Abbreviations

GRoWE: Guide for rehabilitation workforce evaluation
HLMA: Health labour market analysis
RCF: Rehabilitation Competency Framework
WHO: World Health Organization

## References

[1] World Health Organization (2021). Rehabilitation Competency Framework.

[2] Mills J-A, Cieza A, Short SD, Middleton JW (2021). Development and validation of the WHO Rehabilitation Competency Framework: a mixed methods study. Arch Phys Med Rehabil.

[3] Bickenbach J, Sabariego C, Stucki G (2021). Beneficiaries of rehabilitation. Arch Phys Med Rehabil.

[4] Boggs D, Polack S, Kuper H, Foster A (2021). Shifting the focus to functioning: essential for achieving Sustainable Development Goal 3, inclusive Universal Health Coverage and supporting COVID-19 survivors. Glob Health Action.

[5] Stucki G, Bickenbach J, Gutenbrunner C, Melvin J (2018). Rehabilitation: the health strategy of the 21st century. J Rehabil Med.

[6] Bright T, Wallace S, Kuper H (2018). A systematic review of access to rehabilitation for people with disabilities in low- and middle-income countries. Int J Environ Res Public Health.

[7] Kamenov K, Mills JA, Chatterji S, Cieza A (2019). Needs and unmet needs for rehabilitation services: a scoping review. Disabil Rehabil.

[8] Jesus TS, Hoenig H (2019). Crossing the global quality chasm in health care: where does rehabilitation Stand?. Arch Phys Med Rehabil.

[9] Commission on Health Employment and Economic Growth. Working for health and growth: investing in the health workforce. Report of the High‐Level

[10] World Health Organization (2016). Commission on health employment and economic growth.

[11] Coates A, Fuad A-O, Hodgson A, Bourgeault IL (2021). Health workforce strategies in response to major health events: a rapid scoping review with lessons learned for the response to the COVID-19 pandemic. Hum Resour Health.

[12] World Health Organization (2023). Guide for rehabilitation workforce evaluation.

[13] Campbell J, Mills J-A (2022). Health systems and policy research needed to strengthen the rehabilitation workforce. Bull World Health Organ.

